# Metabolomics as a promising tool for early osteoarthritis diagnosis

**DOI:** 10.1590/1414-431X20176485

**Published:** 2017-09-21

**Authors:** E.B. de Sousa, G.C. dos Santos, M.E.L. Duarte, V. Moura, D.P. Aguiar

**Affiliations:** 1 Divisão de Pesquisa, Instituto Nacional de Traumatologia e Ortopedia Jamil Haddad, Rio de Janeiro, RJ, Brasil; 2 Programa de Biologia Celular e do Desenvolvimento, Instituto de Ciências Biomédicas, Universidade Federal do Rio de Janeiro, Rio de Janeiro, RJ, Brasil; 3 Centro Nacional de Biologia Estrutural e Bioimagem, Universidade Federal do Rio de Janeiro, Rio de Janeiro, RJ, Brasil; 4 Instituto Estadual do Cérebro Paulo Niemeyer, Rio de Janeiro, RJ, Brasil

**Keywords:** NMR, Metabolomics, Osteoarthritis, Synovial Fluid

## Abstract

Osteoarthritis (OA) is the main cause of disability worldwide, due to progressive articular cartilage loss and degeneration. According to recent research, OA is more than just a degenerative disease due to some metabolic components associated to its pathogenesis. However, no biomarker has been identified to detect this disease at early stages or to track its development. Metabolomics is an emerging field and has the potential to detect many metabolites in a single spectrum using high resolution nuclear magnetic resonance (NMR) techniques or mass spectrometry (MS). NMR is a reproducible and reliable non-destructive analytical method. On the other hand, MS has a lower detection limit and is more destructive, but it is more sensitive. NMR and MS are useful for biological fluids, such as urine, blood plasma, serum, or synovial fluid, and have been used for metabolic profiling in dogs, mice, sheep, and humans. Thus, many metabolites have been listed as possibly associated to OA pathogenesis. The goal of this review is to provide an overview of the studies in animal models and humans, regarding the use of metabolomics as a tool for early osteoarthritis diagnosis. The concept of osteoarthritis as a metabolic disease and the importance of detecting a biomarker for its early diagnosis are highlighted. Then, some studies in plasma and synovial tissues are shown, and finally the application of metabolomics in the evaluation of synovial fluid is described.

## Introduction

The definition of osteoarthritis (OA) was recently updated by the Osteoarthritis Research Society International to "*a disorder involving movable joints characterized by cell stress and extracellular matrix degradation initiated by micro- and macro-injury that activates maladaptive repair responses including pro-inflammatory pathways of innate immunity*" (1). The disease involves cartilage degeneration, intraarticular synovial inflammation, and subchondral bone thickening (2). These structural joint modifications impact both labor and leisure activities. Hip and knee OA are the leading causes of disability (3).

In 2008, it was estimated that around 27 million people had clinical OA in the United States (4). Projections estimate that by 2030, there will be a 174% increase in hip and 673% increase in knee arthroplasties (5).

This rise demands actions regarding both OA prevention and treatment, due to its impact on public health and quality of life. If molecular markers of OA before the disease becomes irreversible could be identified (6), these statistics could be improved.

### Osteoarthritis pathophysiology

OA is no longer considered only a mechanical "wear and tear" disease (7), as metabolic components have also been involved in its genesis (8,9). One example is the metabolic syndrome, characterized by the triad of hypertension, dyslipidemia, and diabetes, which is associated with low-grade systemic inflammation that triggers OA development by subchondral bone ischemia, articular cartilage damage, and synovial endothelium dysfunction (10).

OA symptoms involve both pain and joint stiffness, which are associated with osteophytes formation, ligamentous laxity, and muscle weakening (11). Diagnosis is clinical, but imaging is useful to determine the severity of the disease, monitor its progression, exclude primary causes, and to establish whether the best treatment is conservative or surgical. Laboratory tests, including blood and synovial fluid analysis, are helpful in evaluating associated conditions (12).

Still today, no treatment has been able to delay, prevent, slow, or reverse the disease. Conservative treatment of OA involves pharmacological and non-pharmacological options. Non-pharmacological treatment consists of a pool of measures including diet for weight loss, physical therapy, and exercise (13). Pharmacological treatment is based on analgesics, anti-inflammatories, and chondro-protective drugs to relieve pain and improve function.

For the cases where conservative measures fail, surgical treatment is indicated depending on the disease severity (14). Arthroscopic debridement and osteotomy may be performed depending on the patient's clinical and radiological evaluation (15). Arthroplasty continues to be the most successful procedure for the treatment of OA, often achieving patient satisfaction (16). Recently, joint distraction has been described to improve clinical results in patients with knee OA for at least 2 years, besides showing positive results in cartilage structure (17).

On the other hand, cell therapies are being postulated as future therapeutic options. Such studies focus mainly on mesenchymal stem cells that originate from different sources, such as bone marrow, adipose tissue and, recently, synovial fluid and the synovial membrane (18–21).

### Osteoarthritis biomarkers

A biomarker can be defined as an objectively measured characteristic, evaluated as an index of normal biological activity, pathological conditions or even responses to pharmacological treatment (22). Biomarkers may help identify early degradation in degenerative diseases, including OA (21), and can be used for decision making in clinical practice (1).

Despite all the efforts in the search for an OA biomarker, only few of the discovered ones have a clinical application. Most of these biomarkers arise from the tissues that suffered the metabolic alterations due to OA (23).

C telopeptide fragment of collagen type II (CTX-II) is one of the most studied fragments. Its concentration seems to be higher in the synovial fluid of patients with early OA when compared to healthy individuals (23). Urinary CTX-II is the most frequently studied biomarker of matrix destruction since its baseline levels predicted pain and radiographic progression in patients with knee OA over a period of 4 years (1,24,22). However, in patients with anterior cruciate ligament and/or meniscal tear, CTX-II is also increased (23).

Cartilage oligomeric matrix protein (COMP) is a biomarker of matrix production and differentiation (24). Deamidated COMP was associated with hip OA severity, while total COMP was associated with knee OA grade (22,25). COMP levels seem to be useful as a marker of hip and knee OA incidence, instead of its progression (24).

C-reactive protein (CRP) is an acute phase protein used as a biomarker for inflammation (23,24). Elevated serum CRP indicates disease activity (22,23). CRP was also related to knee OA incidence and progression (1,24). Unfortunately, the prognostic value of CRP is still inconclusive (24).

### Biomarkers and metabolomics

Global metabolic profiling, called metabolomics, has the potential to identity novel biomarkers that could help elucidate OA diagnosis and could function as targets for the development of new drugs aimed at treatment of OA (26).

Metabolomics involves the study of the metabolites present in a biological system (27), allowing analysis of the organism response to certain environmental stimuli (28). It represents an emerging field and has the potential of detecting 40-150 metabolites in a single spectrum by nuclear magnetic resonance (NMR) or mass spectroscopy (MS) techniques. Considering that living systems are dynamic and complex, a metabolite can participate in a huge number of pathways. Thus, the basis for metabolomics is that a pathological state may alter the concentration of a specific set of metabolites, which can then be targeted as a biomarker of a disease (28). Different forms of arthritis, such as rheumatoid arthritis (29) and septic arthritis (30), have been discriminated from other inflammatory arthritis by metabolomics. In this review, our focus is OA because its pathophysiology is different from other kinds of arthritis and because it lacks a specific biomarker.

High-resolution NMR is a reproducible and reliable non-destructive analytical method. However, MS is more destructive than NMR, but more sensitive and, due to the chemical derivations necessary for the analysis, MS is less reproducible. Both NMR and MS are useful for biological fluids, cells, or tissue extractions. The most commonly investigated biofluids are urine, blood plasma, serum, and synovial fluid (SF), all of which can be obtained with minimal invasion (27,31). Data obtained is analyzed using multivariate analysis to determine patterns or biomarkers representative of certain disease states (28).

### Metabolomics of biological fluids and tissues in osteoarthritis

Metabolism is similar, at some points, in different species; hence, the use of animal models of the disease and treatment may help in the search for metabolic markers in the joint or serum (31). MS and NMR have been used for biological fluid metabolic profiling in dogs (32), mice (33), sheep (34,35), horse (36), and humans (37–41).

A study using K/BxN mice demonstrated that serum could be used for diagnostic or prognostic testing. In this model, it seemed that nucleic acid metabolism was highly impacted by inflammation, and thus, uracil and xanthine were the two most prominent metabolites identified. Hypoxanthine, uridine, and trimethylamine N-oxide were also detected. The study questioned the importance of choosing relevant biomarkers for OA screening, since metabolites can be regulated by different pathways, sometimes confounding the analysis (33).

In another study involving sheep serum NMR analysis, different metabolic responses were identified in two OA models over time. The group with meniscal destabilization showed increased dimethyl sulfoxide levels after 4 weeks and decreased creatine levels after 12 weeks. In animals that underwent anterior cruciate ligament reconstruction, a decrease in branched chains amino acids associated with an increase in 3-methylhistidine at 4 and 12 weeks was registered. Furthermore, glutamine, creatine and creatinine levels were increased at 12 weeks, suggesting altered muscle metabolism (35).

OA patients were stratified into two subgroups according to the absence or presence of joint effusion. Urinary histidine was higher in the former, while urinary histamine was higher in the latter. Increased activity of the tricarboxylic acid cycle because of an altered metabolism in cartilage and chondrocytes was hypothesized due to an elevated expression of aconitic acid, isocitric acid, and citric acid in the urine of OA patients. The same analysis showed a reduced excretion of glutamine, suggesting impairment of energy metabolism in chondrocytes (38).

A recent study involving metabolomic analysis of urine samples of patients with knee OA suggested that measurement of metabolites could be useful in predicting OA progression. Glycolate, hippurate, and trigonelline were important to distinguish the patients prone to OA progression from those who were not prone (42).

Arginine was depleted in the plasma of patients with knee OA. Metabolic targeted profiling also identified five other metabolites associated with knee OA, but arginine was the most significant one. The authors believe that arginine depletion is related to the over activity of the arginine to ornithine pathway, which leads to an imbalance between cartilage degradation and repair. It is also pointed out that arginine is the biomarker identified as having the greatest sensitivity and specificity to discriminate patients with knee OA. However, further longitudinal studies were suggested to examine this affirmation (43).

Another study used samples collected from discarded synovial tissue from patients who underwent either total knee replacement or arthroscopy. These samples were placed in tubes containing Dulbeccos's modified eagle medium (DMEM) culture media and 105 different metabolites were identified across all conditioned media samples. Seven were higher in the end-stage OA group compared to no/early stage group (pro-hydroxyproline, acetylcarnitine, myo-inositol, N-acetylornithine, succinate, glutamine and urea). Among the global metabolic profiling, metabolites related with collagen degradation, amino acid catabolism, energy metabolism, and lipid and carbohydrate metabolism were significantly different in patients with end-stage OA when compared to patients with early or no OA, suggesting its potential use as biomarkers (39).

One important component of osteoarthritis pathophysiology is subchondral bone sclerosis. Hence, the metabolic profiling of subchondral bone was performed and 68 metabolites were found significantly altered in the sclerotic subchondral bone compared to non-sclerotic subchondral bone. Taurine, L-carnitine and glycerophospholipids are involved in the pathogenesis of subchondral bone sclerosis. The results suggest that the sclerotic subchondral bone intra-cellular environment might be more hypoxic and acidotic compared with the non-sclerotic subchondral bone. Beta-alanine and L-carnitine were found to have an effect in the raise of energy consumption (44).

In the last decade, OA has been associated with metabolic syndrome (8,45). Metabolomic analysis of synovial fluid and plasma of OA patients with and without diabetes compared to healthy controls has been performed. Metabolomic analysis revealed that phosphatidylcholine acyl-alkyl C34:3 and phosphatidylcholine acyl-alkyl C36:3 are present in OA and in diabetes patients. This demonstrates that phosphatidylcholine metabolism is associated with OA and diabetes mellitus (46). Plasmatic lysophosphatidylcholine to phosphatidylcholine ratio was identified as a potential new OA biomarker for predicting advanced knee OA. Besides, it was confirmed that BCAAs to histidine ratio was associated with knee OA (47).

The advantage of sampling SF instead of serum in the case of OA is that the breakdown products of cartilage would be detected first in SF (34). However, other biological fluids continue to be investigated since there is still no OA biomarker today.

### Metabolomics of synovial fluid and osteoarthritis

Recently, SF has been studied because it is in contact with all the tissues in the synovial joint, allowing a perfect analysis of the whole joint (48). However, the heterogeneity of the disease makes it difficult to establish a suitable biomarker (35). Hence, the use of metabolomics for the study of osteoarthritic synovial fluid would help in providing a panel of biomarkers that could be used both for diagnosis and for therapeutic management (49).

Several synovial fluid metabolomics studies described different metabolites associated with OA (Table 1), but metabolomic studies paired with healthy synovial fluids are still missing. The complexity among physiological and metabolic domains hinder accurate diagnosis. Metabolic arrays are essential for identification of pathophysiological elements (e.g., cartilage loss, and changes in synovium and surrounding tissues) that appear to have an inconsistent relationship with pain. The knee OA diagnostic umbrella - having grown to assimilate multiple elements of OA pathophysiology - now encompasses a heterogeneous population with a wide range of impairments that do not necessarily imply a specific course of physical therapy treatment. However, the diagnosis of pain phenotypes in knee OA, even in the absence of prognosis or treatment response, may help to guide clinical decision-making. Medical science devoted towards elucidating the molecular causes of OA has gained great weight in medical decisions.

**Table 1.** Main findings of studies involving metabolomic analysis of the synovial fluid of humans and animals with osteoarthritis.

Metabolite	Experimental model	Reference
Lactate, alanine, acetate, N-acetylglucosamine, citrate, creatine/creatinine, glycerol, HDL choline, and α-glucose	Equine	32
Glycerol and hydroxybutyrate	Canine	34
Glycine, serine, creatine, choline hydroxyproline, creatine and proline	Ovine	36
4-methyl-2-oxopenthanoate O-acetylcarnitine, hexanoylcarnitine, N-phenylacetylglycine and ethanolamine	Human	41
Arabitol, glucose, galactose (OA KL1);β-alanine, pyruvate, terephalate (OA KL2)	Human	50

HDL: high-density lipoprotein; OA: osteoarthritis; KL: Kellgren Lawrence classification of osteoarthritis.

A study in horses demonstrated a statistically significant increase in the levels of all metabolites (lactate, alanine, acetate, N-acetylglucosamine, pyruvate, citrate, creatine/creatinine, glycerol, HDL choline, and α-glucose) in OA synovial fluid when compared to healthy controls (36).

In a canine model using denervation-accelerated OA, NMR analysis identified differences in the biochemical profile by comparing normal and osteoarthritic synovial fluids. Glycerol and hydroxybutyrate were increased in the denervated limbs, suggesting that lipolysis plays an important role in joint metabolism. Acetate and N-acetyl group levels were also elevated, indicating degradation of synovial fluid components with the progression of OA severity. Finally, high lactate and low glucose concentrations in denervated-knee fluids suggest that joint denervation aggravates the hypoxic/hypoglycemic intraarticular environment in OA (32).

In an ovine model, NMR was used to access host responses to anterior cruciate ligament reconstruction injury via synovial fluid. Sixty-five metabolites were quantified, six of which could be related to early post-injury degenerative changes: isobutyrate, glucose, hydroxyprolyne, asparagine, serine, and uridine. Most of them were associated with the hypoxic and acidotic conditions of an injured and inflamed joint. However, the metabolite profile was different from sham surgery and could be related to early OA development (34)

NMR was used to compare the spectra of synovial fluid from patients with OA and rheumatoid arthritis (RA), but the results did not help in discriminating OA from RA by the lactate concentrations alone. However, a difference was demonstrated by the lactate/alanine ratio (37).

In addition, in this same study, patients with OA were classified into two different groups (A and B) according to their synovial fluid metabolic profiling. Group A patients had significantly higher acylcarnitine/carnitine ratio than group B, indicating a higher acetyltransferase activity in the first group. On the other hand, although carnitine and acetylcarnitine were significantly lower in group A than in group B, all the other acylcarnitines were higher in group A, indicating differences in fatty acid metabolism between the two groups (40).

Eleven metabolites were identified in human synovial fluid and were important for distinguishing patients with OA. Fructose and citrate are increased, indicating, respectively, a hypoxic condition and high energy requirements. Moreover, the reduced levels of malate suggest dysregulated energy production. O-acetylcarnitine, hexanoylcarnitine, N-phenylacetylglycine, and ethanolamine were also decreased, pointing to higher fatty acid and lipid metabolism in synovial fluid of OA patients (41).

It seems that glycolysis, TCA cycle, metabolism of glycine and serine, and fatty acid and amino acid metabolism pathways may not be adequate for biomarker targeting, since they are common to several inflammatory conditions (31)

Synovial fluid metabolite profile changes in OA are also consistent with the radiographic progression of the disease according to the Kelgreen-Lawrence classification. Besides, twenty-eight metabolites, which included malate, ethanolamide, squalene, glycerol, myristic acid, oleic acid, lanosterol, heptadecanoic acid and capric acid, were higher in late stage OA in comparison to early stage (50).

### Osteoarthritis metabolomics: future trends

Low grade chronic inflammation seems to have a central role in OA pathophysiology (51). Hence, systemic inflammation would predispose individuals to OA development (52,53).

Synovium is essential to joint homeostasis, since its vascular network is responsible for cartilage nutrition and because of the synoviocytes that produce synovial fluid (54). Cartilage damage and altered chondrocytes have a key role in the development of synovial inflammation (51). Synovitis is a typical feature of OA, and the raise in proliferation and number of inflammatory cells requires a modification in cell metabolism from a steady metabolic state to a highly active one (54,55). Hypoxia presents a potential threat to cell function and survival, leading to a shift in mitochondrial respiration favoring glycolysis (55).

Understanding how the components of this systemic metabolic disarrangement interact may help find a shortcut to detect a metabolic target early in OA pathophysiology. In pathological conditions where cell metabolism is compromised, as in OA, there is an increase in the production of pro-inflammatory, pro-catabolic and anti-anabolic factors (51,55).

## Conclusion

Metabolomics seems to be a promising tool for investigation of a biomarker for OA diagnosis, stratification, and treatment. Many biological fluids such as plasma, urine and synovial fluid have been evaluated. However, no useful biomarker has yet been defined. Hopefully, metabolomic arrays will soon lead to OA biomarker identification, allowing researchers to track early disease changes and to create specific antagonists against OA. This improvement may have a massive impact on public health and the economy, minimizing the negative effects of OA in society.
